# Supplementary material for: Structural basis for Scc3-dependent cohesin recruitment to chromatin
Source: eLife. 2018 Aug 15;7:e38356. doi: 10.7554/eLife.38356 (PMC6120753; doi:10.7554/eLife.38356)
Supplement: Supplementary file 1 [file elife-38356-supp1.doc]

| Length of DNA duplex | **Sequence*** |
| --- | --- |
| **15 bp** | 5’ TAAACGAAAGTGAAC 3’ |
| **17 bp**  **19 bp** | 5’ TTTTCAAGGAAACGAAA 3’  5’ TTTTTCAAGGAAACGAAAG 3’ |
| **21bp** | 5’ TCTTTTCAAGGAAACGAAAGT 3’ |
| **32 bp** | 5’ **TGGAAGCCTTTTCAAGGAAACGAAAGTGAACT 3’ |

* dsDNA containing a single 5’ T (top strand) and 5’ A (bottom strand) overhang were used. Only top DNA strand is indicated.

** For fluorescence polarization assays the 5’ end of the top strand of the 32mer was labeled with 6-FAM.
