# Supplementary material for: Structural basis for Scc3-dependent cohesin recruitment to chromatin
Source: eLife. 2018 Aug 15;7:e38356. doi: 10.7554/eLife.38356 (PMC6120753; doi:10.7554/eLife.38356)
Supplement: Supplementary file 2 — All strains are derivatives of W303. [file elife-38356-supp2.doc]

| C3 | *MATa/α* |
| --- | --- |
| C501 | *MATα, SCC1-HA_6_::HIS3* |
| C1073 | *MATa/α, smc3::HIS3/SMC3* |
| C5013 | *MATa/α, scc3::natMX4/SCC3, ura3::SCC3-PK_6_::URA3/ura3* |
| C5014 | *MATa/α, scc3::natMX4/SCC3, ura3::SCC3(K224E, K225E)-PK_6_::URA3/ura3* |
| C5015 | *MATa/α, scc3::natMX4/SCC3, ura3::SCC3(K322E, K330E)-PK_6_::URA3/ura3* |
| C5016 | *MATa, scc3::natMX4, ura3::SCC3-PK_6_::URA3* |
| C5018 | *MATa, scc3::natMX4, ura3::SCC3(K224E, K225E)-PK_6_::URA3* |
| C5020 | *MATa, scc3::natMX4, ura3::SCC3(K322E, K330E)-PK_6_::URA3* |
| C5033 | *MATa/α, scc3::natMX4/SCC3, ura3::SCC3(K224E, K225E, K322E, K330E, K423E, K520E, K669E)-PK_6_:URA3/ura3* |
| C5043 | *MATa/α, scc3::natMX4/SCC3, ura3::SCC3(K423E, K520E, K669E)-PK_6_:URA3/ura3* |
| C5054 | *MATa, scc3::natMX4, ura3::SCC3(K423E, K520E, K669E)-PK_6_:URA3* |
| C5165 | *MATα, SCC1-HA_6_::HIS3, ura3::SCC3-PK_6_::URA3* |
| C5166 | *MATα, SCC1-HA_6_::HIS3, ura3::SCC3(K224E, K225E, K322E, K330E, K423E, K520E, K669E)-PK_6_::URA3* |
