# Supplementary material for: Structural basis for Scc3-dependent cohesin recruitment to chromatin
Source: eLife. 2018 Aug 15;7:e38356. doi: 10.7554/eLife.38356 (PMC6120753; doi:10.7554/eLife.38356)
Supplement: Supplementary file 3 [file elife-38356-supp3.doc]

| cen 4 | TGGTGTGGAAGTCCTAATATCG |
| --- | --- |
|  | TGCATGATCAAAAGGCTCAA |
| cen 5 | AACCTTAACAAATGAAGTAAATTCAAA |
|  | TCAATGTGTTAGTTAAAGCAAAAAGAA |
| cen 6 | GGGCGATGGAAGAGGTAAAGT |
|  | AGCATTAACAACTTCGACAGGT |
| pericen 6 | AAGAAGAATTTAGCGTGGTCAGA |
|  | TCCTTTCTCTCGAGTTTCCGT |
| arm 6 | AGCAACGGATACCAGTCAACT |
|  | TGCTGGTTAACTCGGACTTCA |
